# The Role of MicroRNAs in Arterial Stiffness and Arterial Calcification. An Update and Review of the Literature

**DOI:** 10.3389/fgene.2017.00209

**Published:** 2017-12-12

**Authors:** Sideris Nanoudis, Maria Pikilidou, Maria Yavropoulou, Pantelis Zebekakis

**Affiliations:** ^1^Hypertension Excellence Center, 1st Department of Internal Medicine, AHEPA University Hospital, Thessaloniki, Greece; ^2^Division of Endocrinology and Metabolism, AHEPA University Hospital, Thessaloniki, Greece

**Keywords:** miRNAs, arterial stiffness, vascular calcification, arterial aging

## Abstract

Arterial stiffness is an independent risk factor for fatal and non-fatal cardiovascular events, such as systolic hypertension, coronary artery disease, stroke, and heart failure. Moreover it reflects arterial aging which in many cases does not coincide with chronological aging, a fact that is in large attributed to genetic factors. In addition to genetic factors, **microRNAs (miRNAs)** seem to largely affect arterial aging either by advancing or by regressing arterial stiffness. MiRNAs are small RNA molecules, ~22 nucleotides long that can negatively control their target gene expression posttranscriptionally. Pathways that affect main components of stiffness such as fibrosis and calcification seem to be influenced by up or downregulation of specific miRNAs. Identification of this aberrant production of miRNAs can help identify epigenetic changes that can be therapeutic targets for prevention and treatment of vascular diseases. The present review summarizes the specific role of the so far discovered miRNAs that are involved in pathways of arterial stiffness.

## Introduction

Arterial stiffness is a characteristic feature of normal arterial aging, but is also associated with accelerated cardiovascular disorders including systolic hypertension, coronary artery disease, stroke, and heart failure, irrespective of other risk factors, such as smoking, dyslipidemia, and diabetes mellitus (Laurent et al., 2006; Mitchell et al., 2010; Shirwany and Zou, 2010; Parthenakis et al., 2017). Another age-related process is arterial calcification, which in turn is a known risk predictor that increases morbidity and mortality in cardiovascular diseases (Goettsch and Aikawa, 2013; Jiang et al., 2017). MicroRNAs (miRNAs) are small (19–25 nucleotides long) non-coding RNAs that downregulate their target gene expression post-transcriptionally via mRNA degradation or translational repression (Pan et al., 2010; Vickers et al., 2014). They are widely studied in recent years and their role in cardiovascular dysfunction is to some extent revealed (Pan et al., 2010; Vickers et al., 2014). Identification of over- or underproduction of miRNAs could be therapeutic targets for prevention and treatment of vascular diseases. The objective of this review is to summarize studies of the most prominent miRNAs that affect major pathways of arterial stiffness and calcification. We searched PubMed for original articles on miRNAs and arterial stiffness or arterial calcification and we also used reviews affiliated to the subject and reviewed their references.

## MiRNAs and arterial stiffness

Arterial stiffness results from complicated interactions between multiple components of the vessel wall, including extracellular matrix (ECM) composition, vascular smooth muscle cell (VSMC), and endothelial dysfunction. Collagen and elastin are the most important structural proteins of ECM and key regulators of arterial stiffness as they are responsible for blood vessels' strength and elasticity (Zieman et al., 2005; Wagenseil and Mecham, 2012). Reconstruction of ECM, notably increased levels of aberrant types of collagen and reduction of elastin, appears to be the most important mechanism contributing to arterial stiffness (Zieman et al., 2005; Wagenseil and Mecham, 2012; Fleenor and Berrones, 2015). Matrix metalloproteases (MMPs) are endopeptidases which degrade all kinds of ECM proteins. Thus they play a significant role in arterial stiffness via regulating collagen and elastin levels in ECM (Galis and Khatri, 2002; Zieman et al., 2005; Van Doren, 2015). Moreover, advanced glycation end products (AGEs) contribute to arterial stiffness through cross-linking with ECM proteins, including collagen, which reduces vessel's flexibility (Zieman et al., 2005; Fleenor and Berrones, 2015; Xiaomei et al., 2017). Furthermore, many hormones and cytokines are involved in aortic stiffness, such as **angiotensin II (Ang II)** which promotes arterial stiffness through regulating signaling pathways that result in altered ECM accumulation and increased vascular tone (Rodríguez-Vita et al., 2005; Zieman et al., 2005). Apart from structural abnormalities, VSMC proliferation, migration and calcification, as well as impaired endothelium-dependent dilation through paracrine molecules such as nitric oxide (NO) and endothelin are, also, implicated in the development of arterial stiffness (Zieman et al., 2005; Fleenor and Berrones, 2015). Some of the most prominent miRNAs that affect arterial stiffness through the mechanisms mentioned above are described in the subsequent sections (see Table 1).

**Table 1:** miRNAs influencing biological pathways of arterial stiffness.

**miRNA**	**Arterial stiffness**	**Function**
**miRNAs AFFECTING EXTRACELLULAR MATRIX PROTEINS**		
mir-181b	Decreases	Inhibits TGF-β signaling and ECM production
mir-599	Decreases	Inhibits TGF-β signaling and ECM production
mir-145	Decreases	Inhibits TGF-β signaling and ECM production
mir-29	Decreases	Supresses collagen I, III and IV, but also elastin levels
**miRNAs AFFECTING CYTOSKELETAL PATHWAYS, METALLOPROTEINASES, NITRIC OXIDE, AND ADHESION MOLECULES**		
mir-203	Increases	Defective Src-dependent cytoskeletal remodeling
mir-765	Increases	Reduces apelin and eNOS activity and increases MMP-2, MMP9
mir-155	Increases	Inhibits eNOS activity
mir-1185	Increases	Upregulates VCAM-1 and E-Selectin
mir-126, mir-223	Decrease	Inhibit VCAM-1 and ICAM-1
**miRNAs INDUCING ARTERIAL STIFFNESS THROUGH ANGIOTENSIN II**		
mir-21	Increases	Ang II increases mir-21 through osteopontin and mir-21 increases fibroblast survival and ECM deposition
mir-19b	Decreases	Ang II represses mir-19b and upregulates CTGF
mir-181a	Decreases	Inhibits Ang II-induced osteopontin expression
mir-130a	Increases	Ang II upregulates mir-130a and VSMC proliferation
mir-155	Decreases	Inhibits Ang II-induced VSMC proliferation

*ECM, extracellular matrix; TGF-b, transforming growth factor-b; MMP, matrix metalloproteinase; eNOS, endothelial nitric oxide synthase; Ang II, angiotensin II; CTGF, connective tissue growth factor*.

## MiRNAs affecting extracellular matrix proteins

**Figure 1:**
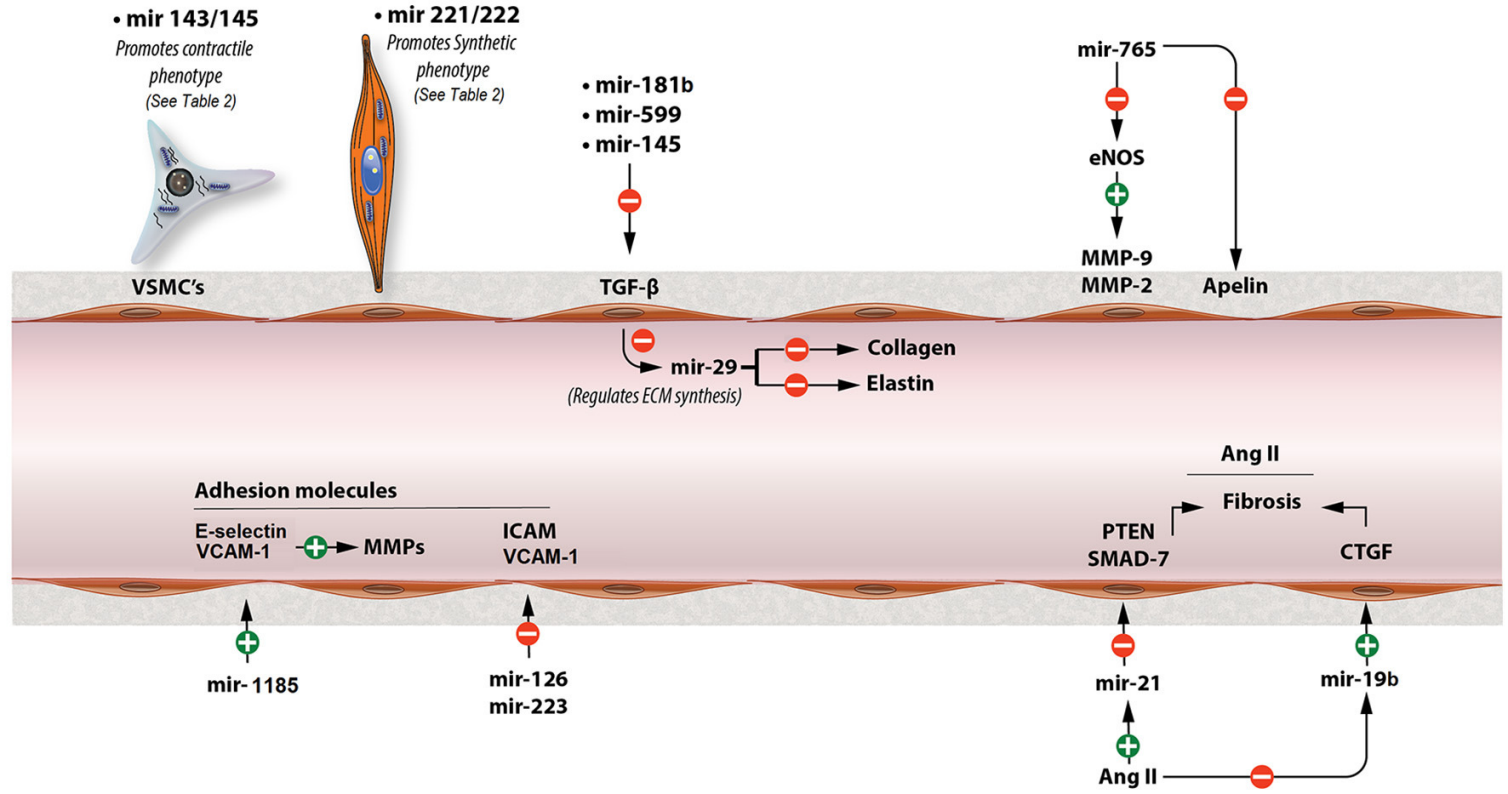
Major miRNAs that contribute to the pathogenesis of arterial stiffness. This figure shows the mechanisms of major miRNAs involved in arterial stiffness through modulating TGF-β signaling, Ang II, adhesion molecules, MMP activity, and VSMC phenotypic switch. ECM, extracellular matrix; TGF-b, transforming growth factor-b; MMP, matrix metalloproteinase; eNOS, endothelial nitric oxide synthase; Ang II, angiotensin II; CTGF, connective tissue growth factor.

**MiR-181b**, which seems to decrease with age, plays critical role in ECM remodeling and regulating vascular stiffness and systolic blood pressure (Hori et al., 2017). Chronic down-regulation of miR-181b expression with age was associated with activation of TGF-β (transforming growth factor-beta) signaling in the VSMCs (Hori et al., 2017). TGF-β can initiate multiple effects in the vessels including phenotypic modulation of the VECs (vascular endothelial cells) and VSMCs (Hori et al., 2017). It is also associated with induction of gene expression such as collagen I and III, stimulating the production of ECM (Hori et al., 2017). Furthermore, it also stimulates plasminogen activator-inhibitor production, which inhibits breakdown of ECM, resulting in increased ECM (Hori et al., 2017). **MiR-181b** inhibits TGF-β signaling pathway by directly inhibiting TGF-βi (transforming growth factor, beta induced) which encodes a protein that interacts with collagen playing a role in cell-collagen interactions (Hori et al., 2017). Similarly, **miR-599** suppresses VSMC proliferation and migration and, also, regulates ECM composition by inhibiting type I, type V collagen and proteoglycan via directly repressing TGF-β2 (Xie et al., 2015). Interestingly, **miR-145** suppresses TGFβ-dependent ECM accumulation and fibrosis by targeting TGFβ receptor II (TGFBR2), while promoting TGFβ-induced smooth muscle cell differentiation (Ning et al., 2015). It seems that a specific miRNA has a selective effect on TGFβ signaling and may facilitate unique downstream events (Ning et al., 2015). Increased expression of a miR-145 mimic in smooth muscle cells resulted in a decrease in TGFβ readout genes, SERPINE1 (PAI-1), and SMAD7 and led to reduced expression of elastin, collagens, and the matrix crosslinking genes, Lox (*Lysyl* oxidase) and Lox1 (Ning et al., 2015). On the other hand, both TGFβ signaling and miR-145 promote the differentiated phenotype of VSMC and contractile gene expressions, such as a-SMA (smooth muscle α-actin) and SM22α (Ning et al., 2015; see Figure 1).

**MiR-29** is considered to be one of the most important miRNAs in regulating ECM composition, as it is known to affect the synthesis of many types of collagen, elastin, MMP-2 and other ECM proteins (Kriegel et al., 2012; Zhu et al., 2013). **MiR-29** reduces fibrosis in scleroderma by repressing type I, III, and IV of collagen, while downregulation of miR-29 from TGF-β promotes renal and cardiac fibrosis (Kriegel et al., 2012; Wang B et al., 2012; Zhu et al., 2013). Interestingly enough, however, other studies demonstrated that miR-29 and miR-15 reduce elastin levels during aortic development in the mouse (Ott et al., 2011; Zhu et al., 2013; see Figure 1). Similarly, Zhang et al. showed **elastin** levels could be increased by inhibiting miR-29a, while collagen levels remained unaffected (Zhang et al., 2012).

## MiRNAs affecting cytoskeletal pathways, metalloproteinases, nitric oxide, and adhesion molecules

The miR-203 expression is increased in aged mouse aorta (Nicholson et al., 2016). Overexpression of miR-203 decreases tyrosine kinase Src and attenuates extracellular signal regulated kinase (ERK) signaling resulting in an insufficient vascular smooth muscle Src-dependent cytoskeletal remodeling which leads to increased arterial stiffness (Min et al., 2012; Gao Y. Z. et al., 2014; Nicholson et al., 2016). Furthermore, miR-765 could accelerate arterial stiffness via knocking down apelin (APLN) and enhancing MMP-2 and MMP-9 (Liao et al., 2015). **APLN** reduces vascular tone through antagonizing Ang II and increasing eNOS activity (Anea et al., 2010; Liao et al., 2015). Also, **both miR-765 and miR-155** lower NO levels by direct eNOS gene downregulation regardless **APLN** intervention (Sun et al., 2012; Liao et al., 2015). **MiR-155**, also, mediates TNFα-induced impairment of endothelium-dependent vasodilation (Sun et al., 2012). Moreover, miR-1185 accelerates arterial stiffness though upregulating VCAM-1 (vascular cell adhesion molecule 1) and E-Selectin (Deng et al., 2017). Adhesion molecules increase arterial stiffness by stimulating MMP activity and, also, affecting vascular tone (Galis and Khatri, 2002; Zieman et al., 2005; Deng et al., 2017). Conversely, miR-126 and miR-223 inhibit VCAM-1 and ICAM-1 (intercellular adhesion molecule 1) activities, respectively, regulating vascular inflammation (Harris et al., 2008; Tabet et al., 2014; see Figure 1).

## MiRNAs and arterial stiffness-clinical studies

Arterial stiffness is most precisely measured by pulse wave velocity (PWV; Laurent et al., 2006). In a study, downregulation of **miR-21** was related to decreased levels of arterial stiffness as estimated by PWV in hypertensive patients at baseline and 1 year after antihypertensive treatment (Parthenakis et al., 2017). Interestingly, the effect on arterial stiffness was not correlated with patients' blood pressure levels (Parthenakis et al., 2017).

## MiRNAs inducing arterial stiffness through Ang II

**MiR-21** plays an important role in Ang II-mediated fibrosis pathways (Laurent et al., 2006; Lorenzen et al., 2015). In cardiac fibrosis, osteopontin **(OPN)** that is activated by Ang II upregulates **miR-21** through transcription factor AP-1. **MiR-21**, then, increases fibrosis through enhancing fibroblast activity and ECM accumulation. In this process, **miR-21** downregulates PTEN (phosphatase and tensin homolog) and SMAD7, while increases ERK and AKT signaling pathways (Lorenzen et al., 2015; see Figure 1). Inversely, **miR-124** exerts an antifibrotic role by reducing pulmonary vascular fibroblast activation by repressing its target gene PTBP1 (polypyrimidine tract-binding protein 1) and subsequently affecting PTEN pathway (Wang et al., 2014).

PTEN is, also, the target of miR-19b-mediated cardiac fibroblast proliferation and migration (Zhong et al., 2016). However, in another study it was demonstrated that Ang II downregulated miR-19b expression and upregulated connective tissue growth factor (CTGF) expression in cultured cardiomyocytes via an AT1R-dependent pathway, increasing cardiac fibrosis (Gao S. et al., 2014; see Figure 1). All these effects of Ang II were abolished by telmisartan, which is an AT1R (Ang II type 1 receptor) antagonist (Gao S. et al., 2014). CTGF is associated with fibrosis through regulating ECM arrangement (Finckenberg et al., 2003; Gao S. et al., 2014). Finally, Ang II promotes VSMC proliferation by upregulating miR-130a which, in turn, decreases GAX expression, the growth arrest-specific homeobox which exerts an inhibitory role on VSMCs proliferation (Wu et al., 2011). On the contrary, **miR-155** overexpression was found to inhibit Ang II-induced viability and proliferation of mouse VSMCs by inducing G1 cell cycle arrest and downregulating the expression of AT1R in VSMCs (Yang et al., 2014).

OPN is a protein expessed in many cells, such as osteoblasts and macrophages but it is normally absent from vessel walls. However, under certain conditions, like injury of the wall or presence of atherosclerosis OPN is overexpressed and contributes to the progression of the inflammatory process (Giachelli et al., 1993; Weintraub et al., 2000; Abe et al., 2008; Remus et al., 2013). **MiR-181a** represses Ang II-mediated OPN increased levels in VSMC as well as Ang II-mediated VSMC adhesion to collagen due to decreased OPN and β1 integrin expression (Weintraub et al., 2000; Remus et al., 2013).

## MiRNAs affecting arterial stiffness through vascular smooth muscle cell function

Vascular smooth muscle cell (VSMC) is the most prevalent type of cell in vasculature and exert multiple functions influencing arterial stiffness (Sehgel et al., 2013, 2015; Fleenor and Berrones, 2015). They are essential regulators of vascular tone through modulating vasoconstriction and vasodilation (Sehgel et al., 2015; Xu F. et al., 2015). Their main characteristic that contributes to the pathogenesis of arterial stiffness is their ability to switch between two distinguishing phenotypes, a contractile (differentiated) and a proliferative (dedifferentiated) form (Davis-Dusenbery et al., 2011b; Kee et al., 2014). The contractile phenotype is defined as a quiescent state and is characterized of reduced ECM arrangement, suspended proliferation and migration and the expression of some marker genes, such as smooth muscle a-actin (a-SMA), SM22a, smooth muscle myosin heavy chain (MYH11), and myosin light chain kinase (MYLC; Davis-Dusenbery et al., 2011b; Kee et al., 2014; Xu F. et al., 2015). Conversely, dedifferentiated or synthetic phenotype induces VSMC proliferation, migration, and ECM accumulation resulting in an increase in arterial stiffness (Davis-Dusenbery et al., 2011b; Kee et al., 2014). There seems to be a plethora of miRNAs associated with VSMC proliferation, migration and phenotypic switch in literature and the most prominent of them are listed below (see Table 2).

**Table 2:** Role of miRNAs in VSMC phenotypic switch.

**miRNA**	**Synthetic phenotype**	**Contractile phenotype**	**Function**
mir-143/145	−	+	TGF-β and BMP activate mir-143/145, which in turn downregulate KLF4 and CD40
mir-133	−	+	Decreases KLF4
mir-1	−	+	Decreases KLF4 and Pim-1
mir-24	+	−	Represses Trb3 and decreases TGF-β and BMP
mir-26a	+	−	Supresses SMAD1 and TGF-β
mir-541	+	−	Represses IRF7
mir-96	+	−	Represses Trb3. Downregulated by BMP4
mir-221/222	+	−	Inhibit p27, p57, c-kit and repress myocardin
mir-223	+	−	Represses myocardin by downregulating mef2c
mir-195	−	+	Represses Cdc42, CCND1, FGF1, IL-1b, IL-6, IL-8
mir-10a	−	+	Represses HDAC4
mir-21, mir-146a	+	−	Inhibit Notch/Jag1 pathway
mir-21	+	−	Inhibits tropomyosin 1
mir-18a-5p	−	+	Represses syndescan4 and increases SMAD2
mir-23b	−	+	Supresses urokinase-type plasminogen activator, SMAD3, FoxO4, MMP-9
mir-663	−	+	Reduces MMP-9
mir-25	−	+	Represses CDK6
mir-142-5p	+	−	Downregulates BTG3 and increases cyclin D3
mir-365	−	+	Decreases cyclin D1
mir-638	−	+	Decreases cyclin D1 and NOR1
mir-141, mir-490-3p	−	+	Repress PAPP-A
mir-155	+	−	Downregulates eNOS expression
mir-135b-5p, mir-499a-3p	+	−	Repress mefc2
let-7d, mir-15b/16	−	+	Decrease KRAS and YAP

*TGF-β, transforming growth factor-b; BMP, bone morphogenetic protein; KLF4, Krüppel-like factor-4; Trb3, Tribbles-like protein-3; IRF7, Interferon regulatory factor 7; HDAC4, histone deacetylase 4; BTG3, B cell translocation gene 3; PAPP-A, Pregnancy-associated plasma protein-A; eNOS, endothelial nitric oxide synthase; mefc2, myocyte enhancer factor 2C*.

## Critical role of BMP, TGF-b, and PDGF-BB

Bone morphogenetic protein (BMP) and transforming growth factor-b (TGF-b) are the major signaling pathways that enhance the contractile phenotype, while, on the other hand, platelet-derived growth factor-BB (PDGF-BB) is the primary pathway inducing the proliferative phenotype (Chan et al., 2010). **MiR-24**, after induced by PDGF-BB, promotes VSMC proliferation through repressing Tribbles-like protein-3 (Trb3), which in turn downregulates SMAD transporters and BMP-TGF-b pathways (Chan et al., 2010). Similarly, miR-26a downregulates TGF-b and promotes VSMC proliferation and migration through suppressing the expression of SMAD-1 (Leeper et al., 2011). Inhibition of **miR-26a**, accelerated VSMC differentiation with a pronounced rise in both MYH11 expression and a-SMA and, also demonstrated increased levels of SMAD-1 and SMAD-4 inhibiting VSMC proliferation and migration (Leeper et al., 2011). Furthermore, PDGF-BB increases the expression of miR-541, which, in turn, promotes VSMC proliferation and invasion by directly repressing IRF7 (Interferon regulatory factor 7; Yang et al., 2016). Also, PDGF-BB can stimulate HIF-1a (hypoxia inducible factor-1a) expression to induce miR-21 upregulation which then inhibits TPM1 expression promoting VSMC proliferation (Wang et al., 2011). On the other hand, low levels of miR-96, which is negatively regulated by BMP4, increase Trb3 and promotes the contractile phenotype (Kim et al., 2014). **MiR-96** is modulated only by BMP signaling and is not affected by PDGF-BB (Kim et al., 2014).

## KLF4 and myocardin

CArG box is a part of DNA that controls the expression of gene markers promoted by the contractile phenotype. Serum response factor (SRF) and myocardin **(Myocd)** promote contractile gene expression via enhancing CArG box activity, while Krüppel-like factor-4 (KLF4) has the opposite effect (Davis-Dusenbery et al., 2011a). TGF-β and BMP4-mediated upregulation of miR-143 and miR-145 through the CArG box downregulate KLF4 which leads to activation of contractile genes and induction of contractile phenotype (Cordes et al., 2009; Xin et al., 2009; Davis-Dusenbery et al., 2011a; Zhang et al., 2016; see Figure 1). **MiR-143/145** are considered to be among the most important miRNAs affecting VSMC phenotype switch by regulating crucial transcription factors, including KLF4 and Myocd. Moreover, **miR-133** indirectly decreases the activity of KLF4 via downregulating the transcription factor Sp-1 (Torella et al., 2011). Suppression of CD40, a type I transmembrane glycoprotein receptor, is another mechanism through which miR-145 contributes to the contractile phenotype of VSMC and inhibits TNF-α -mediated VSMC proliferation (Guo et al., 2016). Finally, miR-143/145 deficiency negatively regulate vascular tone through impaired vasodilation which is considered to result from increased VSMC switch to the dedifferentiated phenotype (Noratal et al., 2012). Similarly to **miR-143/145**, **miR-1** represses KLF4 and promotes VSMC differentiation from mouse embryonic stem cells (ESC; Xie et al., 2011). Also, Myocd induces miR-1 expression in human VSMCs and, on the other hand, miR-1 mediates **Myocd**-dependent inhibition of VSMC proliferation partly through the down-regulation of Pim-1, a kinase, which promotes the synthetic phenotype (Chen et al., 2011). Conversely, miR-221/222 promote proliferation of VSMCs partly by inhibiting p27(Kip1) and p57(Kip2) which inhibit VSMC proliferation (Liu et al., 2009, 2012; Chistiakov et al., 2015; see Figure 1). Moreover, PDGF-induced miR-221 has the same effect through another mechanism including downregulation of c-kit and Myocd (Davis et al., 2009). The expression of Myocd is also repressed by miR-223 via downregulating mef2c (Rangrez et al., 2012).

## MiRNAs affecting VSMC function through NF-κB pathway

**Myocd** induces differentiation and contractile phenotype of VSMCs and is, also, associated with inhibition of VSMC proliferation through downregulating NF-κB (nuclear factor kappa-light-chain-enhancer of activated B cells) (p65)-dependent cell cycle progression (Tang et al., 2008; Chen et al., 2011). Similarly, miR-195 promotes contractile phenotype by suppressing Cdc42, CCND1, FGF1 and proinflammatory biomarkers, such as IL-1b, IL-6, and IL-8, which are also implicated in NFkB and p38 MARK (mitogen-activated protein kinases) pathway (Wang Y. S. et al., 2012). Another study, however, demonstrated that miR-10a enhances VSMC differentiation from ESCs through NF-κB-miR-10a-HDAC4 signaling pathway (Huang et al., 2010). miR-10a mediates VSMC differentiation through repressing HDAC4 (histone deacetylase 4) and preventing its antimyogenic effects, as HDAC4 overxpression reduces VSMC markers, including MYH11 and a-SMA (Huang et al., 2010).

## MiRNAs targeting MMP-9, TNF-a, cyclin D, PPAP-A, and eNOS activity

**MiR-23b** inhibits VSMC switch to proliferative phenotype via suppressing urokinase-type plasminogen activator, SMAD3 and transcription factor forkhead boxO4 (FoxO4; Iaconetti et al., 2015). Through downregulating FoxO4, **miR-23b** might, also, reduce MMP-9 (matrix metalloproteinase 9) levels and TNF-a induced VSMC migration (Iaconetti et al., 2015). MMP-9 is also reduced by **miR-663** by inhibiting JunB/myosin light chain 9 pathway which results in increasing VSMC contractile phenotype and marker genes (Korff et al., 2013; Li et al., 2013b). Also, miR-25 inhibits VSMC proliferation via directly downregulating the CDK6 gene, a cell cycle regulator, and, on the other hand, **miR-25** is repressed in TNF-a-mediated VSMC dedifferentiated phenotypic switch (Qi et al., 2015). **MiR-142-5p** promotes the synthetic phenotype by supressing B cell translocation gene 3 (BTG3) that results in increased levels of cyclin D3 and other cell cycle related genes (Kee et al., 2013). On the other hand, **miR-365** promotes contractile phenotype through negatively regulating cyclin D1 (Zhang et al., 2014). **MiR-638** has the same effect by targeting the NOR1/cyclin D pathway and repressing the expression of both NOR1 and cyclin D1 which exert a pro-proliferative effect (Li et al., 2013a). Furthermore, **miR-141** and **miR-490-3p** inhibit VSMC proliferation through repressing PAPP-A (Pregnancy-associated plasma protein-A; Sun et al., 2013; Zhang Y. et al., 2015). PAPP-A is a metalloproteinase correlated with VSMC proliferation via increasing the proteolysis of IGF-binding protein-4 (IGFBP-4; Sun et al., 2013; Zhang J. et al., 2015). Apart from regulating nitric oxide (NO) production that results in vascular relaxation, endothelial nitric oxide synthases (eNOS) significantly decreases proliferation and migration of VSMCs and functions as a pro-apoptotic protein (Zhang J. et al., 2015). **MiR-155** promotes the proliferative phenotype through downregulating eNOS expression in VSMCs (Zhang J. et al., 2015).

## MiRNAs targeting notch pathway, syndecan4, MEF2C, KRAS, and YAP

The Notch signaling pathway, which is important in intercellular signaling communication, can, also, affect VSMC phenotypic switch (Cao et al., 2015). **Mir-146a** and miR-21 enhance VSMC proliferation via inhibiting Notch2/Jag1 pathway as they downregulate Notch2 and Jag1, respectively (Cao et al., 2015). **MiR-18a-5p** promotes contractile phenotype and increases a-SMA and SM22α protein amounts by repressing its target gene syndecan4 (Kee et al., 2014). Knockdown of syndecan4 increases **SMAD2** expression in VSMCs, which in turn promotes VSMC differentiation by inducing protein expression and promoter activity of SM22α (Kee et al., 2014). Likewise, let-7d and **miR-15b/16** attenuate VSMC proliferation through decreasing KRAS and YAP expression, respectively (Yu et al., 2011; Xu F. et al., 2015). Both KRAS and YAP positively regulate signaling pathways that result in increased VSMC proliferation and migration (Yu et al., 2011; Xu F. et al., 2015). On the other hand, miR-135b-5p and miR-499a-3p promote VSMCs proliferation and migration by directly repressing MEF2C (myocyte enhancer factor 2C) expression (Xu Z. et al., 2015).

## MiRNAs and arterial calcification

Vascular calcification is highly associated with cardiovascular diseases, such as diabetes mellitus, chronic kidney disease, and atherosclerosis, and increases cardiovascular mortality (Goettsch and Aikawa, 2013). Arterial calcification leads to arterial stiffening and hypertension, due to transdifferentiation of VSMCs to a chondrocyte or osteoblast-like phenotype and elastic fiber degradation (Jiang et al., 2017). Here, we mention the major miRNAs that contribute to arterial calcification (see Table 3).

**Table 3:** Major miRNAs involved in arterial calcification.

**miRNA**	**Arterial calcification**	**Function**
**miRNAs AFFECTING ARTERIAL CALCIFICATION THROUGH METALOPROTEINASES**		
mir-29b-3p	Decreases	Represses MMP-2
**miRNAs AFFECTING ARTERIAL CALCIFICATION THROUGH CALCIUM DEPOSITION AND OSTEOBLAST DIFFERENTIATION**		
mir-29a/b	Decreases	Supresses ADAMTS-7
mir-30b-c	Decreases	Supresses RUNX2
mir-133a	Decreases	Supresses RUNX2
mir-204	Decreases	Supresses RUNX2
mir-205	Decreases	Supresses RUNX2 and SMAD1
mir-32	Increases	Induces BMP2, RUNX2, OPN, MGP, ALP
mir-2861, mir-3960	Increase	Repress HDAC5 and Hoxa2 and increase RUNX2
mir-297a	Decreases	Downregulates FGF23
**miRNAs AFFECTING ARTERIAL CALCIFICATION THROUGH SMOOTH MUSCLE CELL OSTEOGENIC TRANSDIFFERENTIATION**		
mir-125b	Decreases	Supresses osterix
mir-135a	Decreases	Downregulates KLF4/STAT3 pathway
mir-221/222	Increase	Alter Enpp1 and Pit-1 and regulate Pi and PPi levels

*BMP-2, bone morphogenetic protein-2; ADAMTS-7, disintegrin and metalloproteinase with thrombospondin motifs-7; RUNX2, Runt-related transcription factor 2; OPN, osteopontin; MGP, matrix GLA protein; ALP, Alkaline phosphatase; HDAC5, histone deacetylase 5; Hoxa2, Homeobox A2; FGF23, fibroblast growth factor 23; Enpp1, ectonucleotide phosphodiesterase 1; Pit-1, Pi cotransporter-1; Pi, inorganic phosphate; PPi, pyrophosphate*.

## MiRNAs affecting arterial calcification through metaloproteinases (MMP-2)

**Figure 2:**
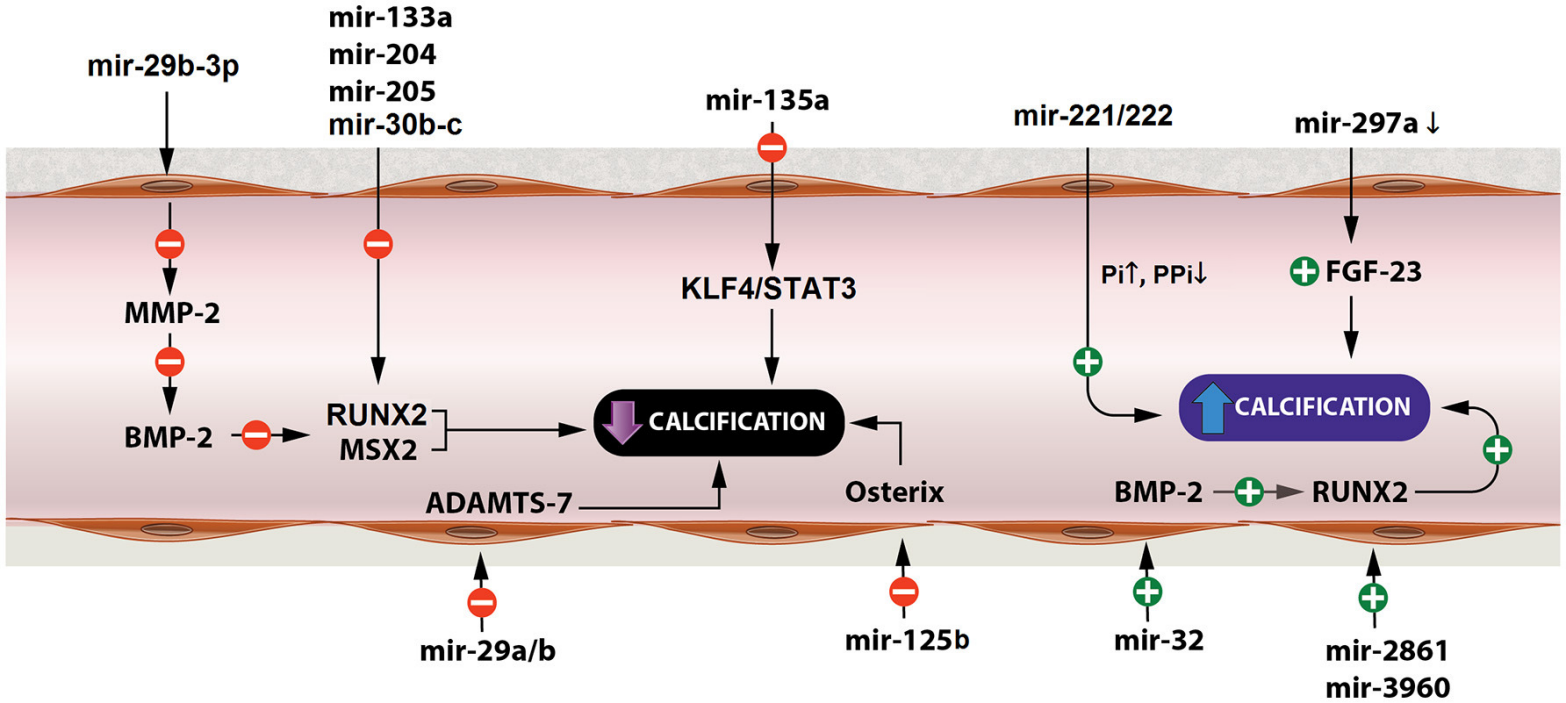
miRNAs affecting arterial calcification. miRNAs play a significant role in arterial calcification by regulating key factors of the calcification process, such as BMP-2, RUNX, ADAMTS-7, osterix, and Pi levels. BMP-2, bone morphogenetic protein-2; RUNX, Runt-related transcription factor 2; ADAMTS-7, disintegrin and metalloproteinase with thrombospondin motifs-7; Pi, inorganic phosphate.

One of the pathways of vascular calcification is through dysregulation of matrix metalloproteinase-2 (MMP-2) which is a gelatinase that has an important role in matrix degradation and vascular remodeling (Jiang et al., 2017; see Figure 2). Upregulation of MMP2 expression and activity is shown in VSMCs and animal calcification models, which mediate elastin degradation, resulting in the production of soluble elastin peptides and stimulation of TGF-β1 signaling pathway from the ECM of vessel walls (Jiang et al., 2017). MMP2 upregulation also contributes to bone morphogenetic protein 2 (BMP2) expression in a β-glycerophosphate-induced VSMC calcification model that could increase **Runx** and **Msx2** expression and promote VSMC calcification (Jiang et al., 2017). MMP2 is a direct target gene of miR-29b-3p with a negative correlation between them (Jiang et al., 2017). Overexpression of miR-29b-3p in rat VSMCs resulted in MMP2 downregulation at the protein level and this could lead to decreased arterial and VSMCs calcification (Goettsch and Aikawa, 2013).

## MiRNAs affecting arterial calcification through calcium deposition and osteoblast differentiation

**MiR-29a/b**, also, decreases VSMC calcification by repressing ADAMTS-7 (disintegrin and metalloproteinase with thrombospondin motifs-7; Du et al., 2012; see Figure 2). ADAMTS-7 mediates VSMC calcification via cartilage oligomeric matrix protein (COMP) degradation and increased levels of BMP2, SMAD proteins and Runx2 which result in increased mineralization (Du et al., 2012). COMP is a glycoprotein that prevents osteochondrogenic transdifferentiation of VSMCs partly by inhibiting BMP2 (Du et al., 2012).

Runx2 is a major transcription factor related to osteoblast transdifferentiation of VSMCs (Balderman et al., 2012). In VSMCs, Runx2 is not expressed under normal conditions but is overproduced in response to procalcifying stimuli, such as inflammation, oxidant stress, and bone morphogenetic protein-2 (BMP-2; Balderman et al., 2012). Runx2 is expressed in osteoblast-like VSMCs and upregulation of Runx2 drives VSMC transition to osteoblast-like cells (Balderman et al., 2012). Furthermore, osteoblast differentiation is, also, strongly associated with BMP-SMAD signaling pathways which subsequently interfere with Runx2 to enhance the overall process of bone formation (Qiao et al., 2014). **MiR-30b-c** suppress Runx2 protein expression preventing VSMC calcification (Balderman et al., 2012). On the other hand, BMP-2 downregulates miR-30b-c and, thereby, increases Runx2 expression promoting calcification of SMCs through a Smad-independent pathway (Balderman et al., 2012). **MiR-133a** and **miR-204** are, also, negative regulators of osteogenic differentiation of VSMCs and inhibit arterial calcification via direct suppression of Runx2 (Cui et al., 2012; Liao et al., 2013). **MiR-205** reduces calcification by targeting SMAD-1, in addition to Runx2, suppressing the expression of both of them (Qiao et al., 2014). Conversely, miR-32 increases mouse VSMC calcification by inducing the expression of BMP2, Runx2, osteopontin (OPN), bone-specific phosphoprotein matrix GLA protein (MGP), and ALP activity (Liu et al., 2017). **MiR-32** enhances Runx2 expression and activity by decreasing PTEN (phosphatase and tensin homolog) levels and subsequently activating the PI3K-Akt (phosphatidylinositol 3-kinase/protein kinase B) signaling pathway in mouse VSMCs (Liu et al., 2017). Likewise, miR-2861 and miR-3960 enhance arterial calcification and osteoblastic formation of VSMCs through repressing histone deacetylase 5 (HDAC5) and Homeobox A2 (Hoxa2), respectively, which, in turn, result in increased levels of Runx2 (Kanzler et al., 1998; Kang et al., 2005; Xia et al., 2015; see Figure 2).

Moreover, fibroblast growth factor 23 (FGF23) modulates phosphorus levels, negatively regulates Klotho and is positively correlated to vascular calcification (Zheng et al., 2016). When Klotho is suppressed (like in rats with vascular calcification) FGF23 could enhance hyperphosphate-induced vascular calcification (Zheng et al., 2016). FGF23 is a potential target of **miR-297a** (Zheng et al., 2016). Low levels of miR-297a upregulate FGF23 and as a consequence suppresses Klotho resulting in further enhancement on vascular calcification (Zheng et al., 2016; see Figure 2).

## MiRNAs affecting arterial calcification through smooth muscle cell osteogenic transdifferentiation

Also, miR-125b modulates arterial calcification by targeting transcription factor SP7 (osterix), since inhibition of endogenous miR-125b in calcified HCASMCs (human coronary artery smooth muscle cells) increases Runx2 and promotes osteoblastic transdifferentiation (Goettsch et al., 2011). Similarly, downregulated miR-135a resulted in elevated levels of osteocalcin and induced calcification (Lin et al., 2016). Moreover, miR-135a seems to be a potential osteogenic differentiation suppressor in senescent VSMCs involving, at least partially, the KLF4/STAT3pathway as it decreases STAT3 expression through directly downregulating KLF4 (Fukuyo et al., 2014; Lin et al., 2016). Other miRNAs that promote arterial calcification and osteoblastic differentiation of VSMCs are miR-221 and miR-222, which have to act cooperatively (Mackenzie et al., 2014). This function is independent of Runx2 and Msx2 and seems to be induced by altered Enpp1 (ectonucleotide phosphodiesterase 1) and Pit-1 (Pi cotransporter-1) expressions that regulate cellular inorganic phosphate (Pi) and pyrophosphate (PPi) levels (Mackenzie et al., 2014; see Figure 2).

## Conclusions

MiRNAs are a novel class of non-coding RNAs that regulate gene expression. Extensive research during the last decade confirmed the association of miRNAs with cardiovascular diseases. MiRNAs seem to play a significant role in arterial stiffness and calcification through modulating critical pathways and molecules such as TGF-β and BMP signaling, Ang II, MMP activity, Runx, and phenotypic switch of VSMC. Thus, they may be used as therapeutic targets or diagnostic markers in the future to decrease arterial stiffness and prevent the development of cardiovascular diseases. However, it is more than obvious that the molecular biology and pathophysiology is very complex. As mentioned above, many miRNAs might have the same target gene (e.g., Runx2 is suppressed by miR-30b-c but enhanced by miR-32), while a single miRNA might exert multiple functions by targeting more than one genes and affecting different pathways with opposing results (e.g., miR-29, miR-19b and their role in fibrosis). Futhermore, miR-145, one of the most important miRNAs in cardiovascular pathophysiology, decreases arterial stiffness by inhibiting TGF-b signaling while, on the contrary, TGF-b activates miR-145 to promote the contractile phenotype of VSMCs and reduce arterial stiffness as well. Targeting TGF-b through miR-145 might have controversial results. To conclude, additional clinical and laboratory research should be continued for the establishment of miRNAs as treatment targets and biomarkers of cardiovascular diseases. Their emerging role is promising and could result in reducing overall cardiovascular morbidity and mortality in the near future.

## Author contributions

SN and MP: Conceived and designed the work, collected the data, and drafted the article; MY: Contributed in drafting the manuscript and critical revision; PZ: Contributed in drafting the manuscript, critical revision, and final approval of the version to be published.

### Conflict of interest statement

The authors declare that the research was conducted in the absence of any commercial or financial relationships that could be construed as a potential conflict of interest.
